# Complications of regional citrate anticoagulation: accumulation or overload?

**DOI:** 10.1186/s13054-017-1880-1

**Published:** 2017-11-19

**Authors:** Antoine G. Schneider, Didier Journois, Thomas Rimmelé

**Affiliations:** 1 Adult Intensive Care Unit, Centre Hospitalier Universitaire Vaudois (CHUV), 46 avenue du Bugnon, 1011 Lausanne, Switzerland; 2 Université de Lausanne, UNIL, Lausanne, Switzerland; 3 Anesthesiology and Intensive Care Medicine, Cochin Hospital, Assistance Publique Hôpitaux de Paris, René Descartes University, Paris, France; 4 Anesthesiology and Intensive Care Medicine, Edouard Herriot Hospital, Hospices Civils de Lyon, Lyon, France; 5 EA 7426 (Université Claude Bernard Lyon 1 – Hospices Civils de Lyon – bioMérieux) “Pathophysiology of Injury-induced Immunosupression – PI3”, Joint Research Unit, Edouard Herriot Hospital, Lyon, France

**Keywords:** Regional citrate anticoagulation, Continuous renal replacement therapy, Acute kidney injury, Citrate accumulation, Complications of therapy, Metabolic alkalosis

## Abstract

Regional citrate anticoagulation (RCA) is now recommended over systemic heparin for continuous renal replacement therapy in patients without contraindications. Its use is likely to increase throughout the world. However, in the absence of citrate blood level monitoring, the diagnosis of citrate accumulation, the most feared complication of RCA, remains relatively complex. It is therefore commonly mistaken with other conditions. This review aims at providing clarifications on RCA-associated acid-base disturbances and their management at the bedside. In particular, the authors wish to propose a clear distinction between citrate accumulation and net citrate overload.

## Introduction

Anticoagulation is required during continuous renal replacement therapy (CRRT) to maintain circuit patency. Heparin has historically been the standard choice for anticoagulation [1]. Unfortunately, in fear of bleeding complications, heparin is often administered at sub-therapeutic doses and frequently interrupted for procedures. The resulting anticoagulation is commonly insufficient, leading to poor filter life [2].

Regional citrate anticoagulation (RCA) is an appealing alternative since it provides excellent anticoagulation within the circuit without increasing the risk of bleeding [2, 3]. In randomized controlled trials [4–6] and meta-analyses [7, 8], RCA has been shown to increase filter life and decrease the rate of complications, therapy interruptions, and costs compared with heparin. RCA has been used in large tertiary centers with very low complication rates [9]. RCA is now recommended as the first line anticoagulation strategy for CRRT in patients without contraindications [10].

Given these recommendations, RCA is likely to be gradually adopted in an increasing number of centers, including smaller and non-academic hospitals with less experience with CRRT. The implementation of RCA requires particularly strict protocols and specific training of both medical and nursing staff. Indeed, unguided RCA might lead to potentially disastrous complications offsetting its potential benefits. Currently published literature might lead to some confusion regarding the interpretation of RCA complications, in particular regarding acid-base derangements.

This viewpoint aims at providing clarifications on citrate-associated acid-base disturbances and their management at the bedside. In particular, the authors wish to propose a clear distinction between citrate accumulation and citrate overload, two intertwined notions, which are commonly confused.

### General principles

#### Principles of citrate anticoagulation

**Fig. 1:**
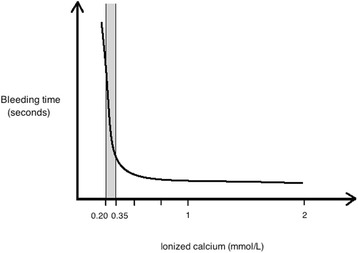
“ON-OFF” anticoagulation effect of ionized hypocalcemia. The *grey zone* corresponds to the area of adequate anticoagulation. Target values indicated are only indicative and depend on the protocol used [12]

Citrate (C_6_H_5_O_7_) is an organic acid. It is commonly used as an anticoagulant as trisodium citrate and, for stored blood products, as acid citrate dextrose (ACD). Citrate anticoagulant properties are related to its high affinity for the divalent calcium ion (Ca^++^). The addition of citrate to blood results in the formation of citrate–calcium complexes (CCC), effectively decreasing the level of ionized free calcium. Ionized magnesium is also chelated by citrate but to a lesser extent. Since calcium is a mandatory co-factor of most enzymes of the coagulation cascade, citrate-mediated decrease in plasma calcium levels below 0.35 mmol/l results in very effective anticoagulation (Fig. 1) [11, 12].

**Fig. 2:**
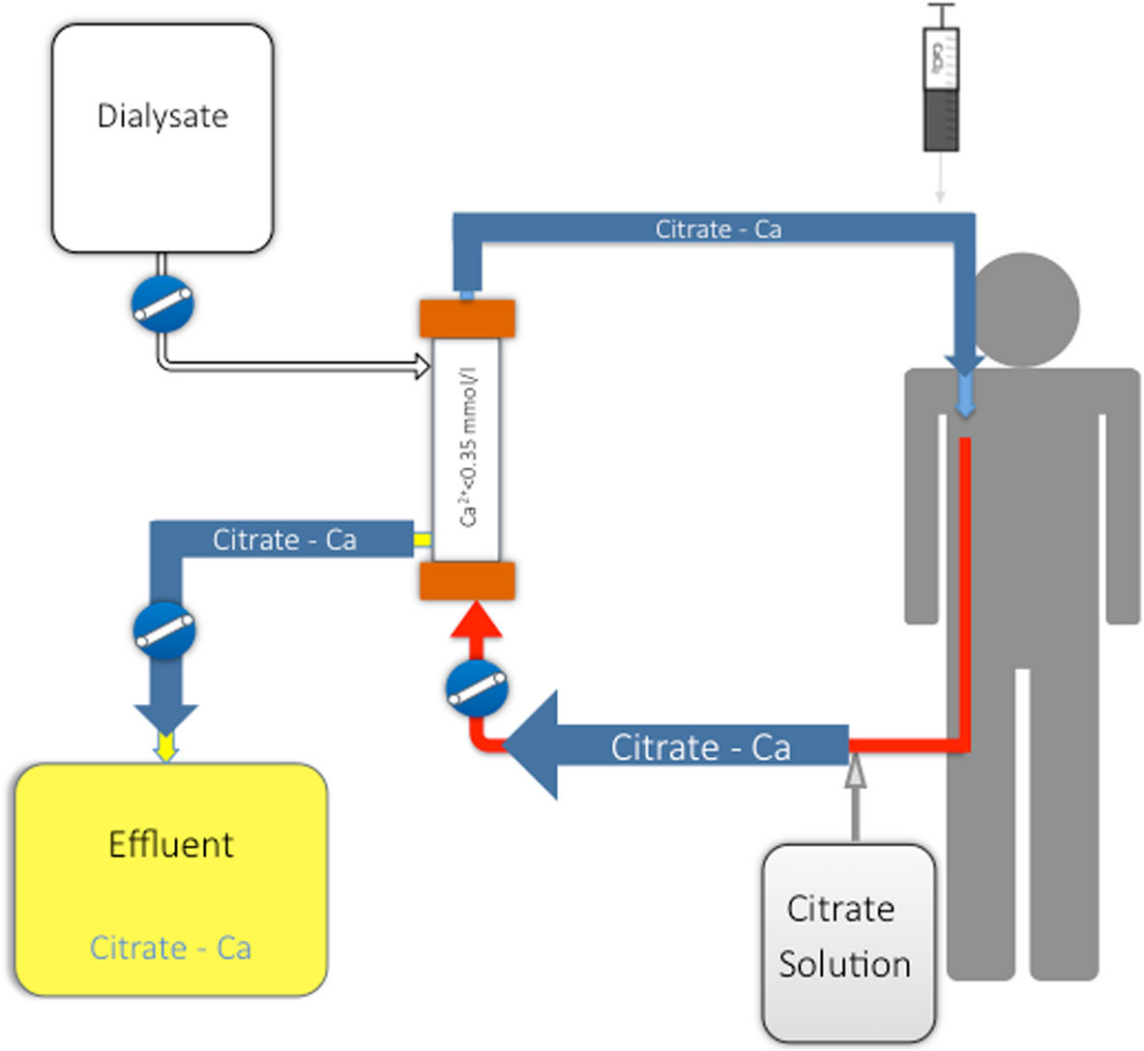
Schematic view of a CRRT circuit with regional citrate administration in CVVHD mode. Alternative modes can be used (postdilution CVVH, combined pre- and postdilution CVVH, CVVHDF, etc.) according to the protocol used. Citrate solution is administered at the beginning of the CRRT circuit. It forms citrate–calcium complexes, which are largely removed from the blood at the level of the filter. Only complexes which are not removed through the hemofilter return to the patient’s blood and need to be metabolized

Numerous protocols for RCA have been proposed and tested [13, 14]. They differ by solution type (ACD, trisodium citrate, diluted citrate solutions) and CRRT modality (continuous veno-venous hemofiltration (CVVH), continuous veno-venous hemodialysis (CVVHD), continuous veno-venous hemodiafiltration (CVVHDF)). All these protocols require pre-filter administration of a citrate solution at the required dose to reach approximately 3 to 4 mmol of citrate per liter of blood in the circuit. Such a dose is usually sufficient to decrease ionized calcium to the target range (0.2 to 0.35 mmol/l according to the protocol used). Post-filter calcium is monitored to ensure adequate anticoagulation and permit citrate dose adjustment according to pre-defined models. In current CRRT machines, citrate administration rate is coupled with blood flow, minimizing the risk of variation in citrate concentration. A calcium chloride solution needs to be administered either at the end of the circuit or directly through a separated central line to compensate for calcium loss in the effluent in the form of CCC (Fig. 2). Calcium reinfusion rate is adjusted according to sequentially measured systemic ionized calcium level (targeting physiological range). After the initiation phase, regular monitoring (every 6 h) of post-filter, systemic, and total calcium levels (with total/ionized ratio calculation) should be performed. Since, according to the composition of dialysate/replacement fluids used, magnesium might also need to be supplemented, daily monitoring of serum magnesium levels is also advisable.

#### Citrate clearance and metabolism

As depicted in Fig. 2, a large portion of CCC is removed through the hemofilter [15]. CCC clearance is very high (up to 60%) due to their low molecular weight (298 Daltons) associated with their high hydrosolubility conferred by the negative charge of a free carboxylate radical. Their sieving coefficient is 1.0. The clearance must be maintained as high as possible to minimize the administration of citrate to the patient. This clearance increases with the dialysate flow (the higher the dialysate flow, the higher the clearance). In convective modes, citrate’s clearance is dependent on filtration flow (the higher the filtration flow, the higher the clearance). CCC which are not removed through the hemofilter return to the patient. They are metabolized in the liver, muscle, and kidney fitting into the Krebs (citric acid) cycle. Under normal conditions, citrate’s half-life is approximately 5 minutes. The process generates energy (2.48 KJ or 593 calories per mmol of citrate), releases sodium as well as calcium ions [16].

#### Citrate and acid-base balance

**Fig. 3:**
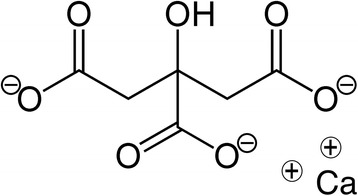
Citrate calcium complex. The distance between calcium’s two positive charges corresponds to the distance between two citrate carboxylate radicals. A carboxylate radical remains unbound, providing residual anionic charge and a *mild* acidic effect. This acidifying effect would be much stronger in vitro, in the absence of ionized calcium

Acid-base consequences of RCA are often reduced to bicarbonate generation by citrate metabolism. Unfortunately, this simplification is inaccurate and correct understanding of citrate’s effect on acid-base balance requires the use of Stewart’s global approach [17–19]. Briefly, according to this approach, blood pH is mainly determined by three variables: PaCO_2_, strong ion difference (SID), and weak acids concentration. Citrate belongs to the weak acid category and its effect should be to markedly acidify a solution. Its three carboxylate radicals have respective pKa values of 5.21, 4.28, and 2.92 at 25 °C) [20]. However, in plasma, unless the calcium level is extremely low (to levels incompatible with life), citrate is only present in the form of CCC. In that form, its acidifying capacity is limited by the binding of ionized calcium to two adjacent carboxylates, leaving only one residual anionic charge (Fig. 3). Circulating CCC therefore lead to mild plasma acidification. Under normal conditions, this effect is negligible since CCC are rapidly cleared from the blood.

However, the acid-base impact of RCA is not limited to the effect of citrate itself. Indeed, the composition and amount of dialysis/substitution fluid used are of major importance. Many citrate *solutions* have a high sodium content (three Na^+^ for one citrate molecule). This net sodium administration tends to increase plasma SID leading to plasma alkalinization.

Overall, when citrate catabolism is normal, RCA leads to *plasma alkalinization*. This alkalinizing effect is maximal with trisodium citrate solutions and less marked with ACD solutions (which have a low sodium content). To some extent, this alkalinization is desirable as it buffers acute kidney injury associated-acidosis and normalizes pH. As discussed in further sections, in some clinical situations where citrate catabolism is markedly impaired, CCC tend to accumulate, generating a mild acidosis.

### Citrate accumulation and alternative diagnoses

Citrate accumulation is a feared and potentially lethal complication of RCA. Fortunately, when a strict protocol is followed, it is rarely encountered [9]. In order to avoid unnecessary therapy interruptions, it is essential for the clinician to distinguish citrate accumulation from other situations resulting in acid-base disturbance during RCA: citrate net overload and insufficient trisodium-citrate delivery. The main differences between these entities are summarized in Table 1.

**Table 1:** Citrate accumulation and alternative diagnoses: summary table

	Citrate accumulation	Citrate net overload	Insufficient trisodium citrate delivery
Mechanism	Incomplete citrate metabolism: persistence of circulating citrate–calcium complexes in the blood	Excess citrate administration relative to buffer requirements	Insufficient alkalotic load administered to the patient to adequately buffer acute kidney injury-associated acidosis
Diagnosis			
Acid-base	Metabolic acidosis	Metabolic alkalosis	Metabolic acidosis
Ca_tot_/Ca_i_ ratio	Increased (>2.5)	Normal (< 2.5)	Normal (< 2.5)
Other	Increased need for calcium substitution Trend for a decreased ionized calcium level	None	None
Appreciation	Potentially lethal (via severe hypocalcemia)	Benign and easy to fix	Benign and easy to fix
Incidence	Rare	Common	Rare
Management	Decrease blood flow or increase dialysate flow rate (if mild) Consider alternative anticoagulation strategy	Decrease blood flow or increase dialysate flow rate	Increase blood flow or decrease dialysate flow rate

#### Citrate accumulation

**Fig. 4:**
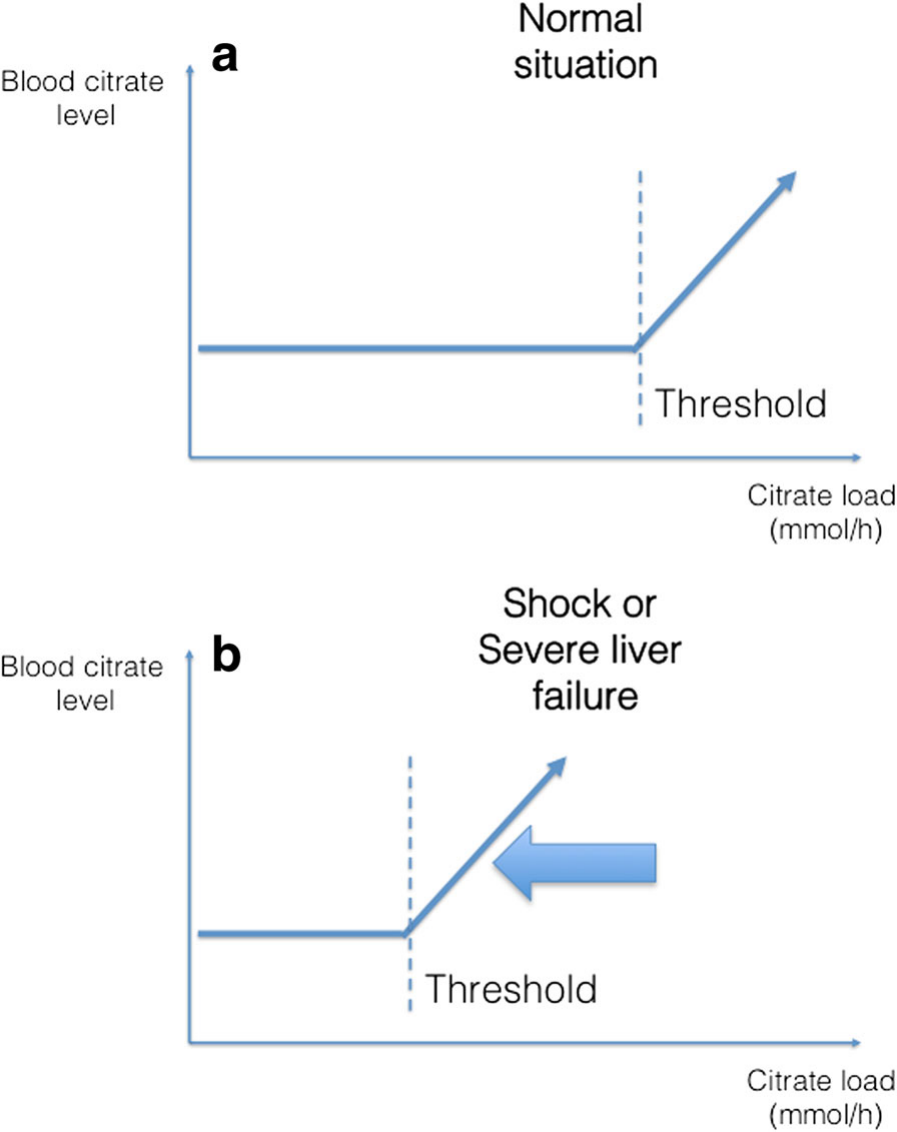
Theoretical relationship between blood citrate level and citrate load. **a** An increase in citrate load is not associated with an increase in blood citrate level until a threshold is reached. This threshold corresponds to the body’s capacity to metabolize citrate. **b** Certain circumstances, such as severe liver failure or circulatory shock, might result in a lower threshold corresponding to a decreased capacity to metabolize citrate (see text)

The body’s capacity to metabolize citrate is saturable (Fig. 4). If citrate administration exceeds this capacity, residual citrate, in the form of CCC remain in blood. In the absence of a routinely available assay for citrate blood level, citrate accumulation can only be suspected through indirect signs. The most reliable sign for citrate accumulation is probably an increased total/ionized calcium (Ca/Ca^++^) ratio. Indeed, an increase in this ratio demonstrates an increase in the serum level of anion-bound calcium, which in the context of RCA is almost synonymous with circulating CCC. A cut-off value of 2.5 is usually recognized as indicative of significant accumulation but a trend towards this value is highly indicative of ongoing accumulation.

**Fig. 5:**
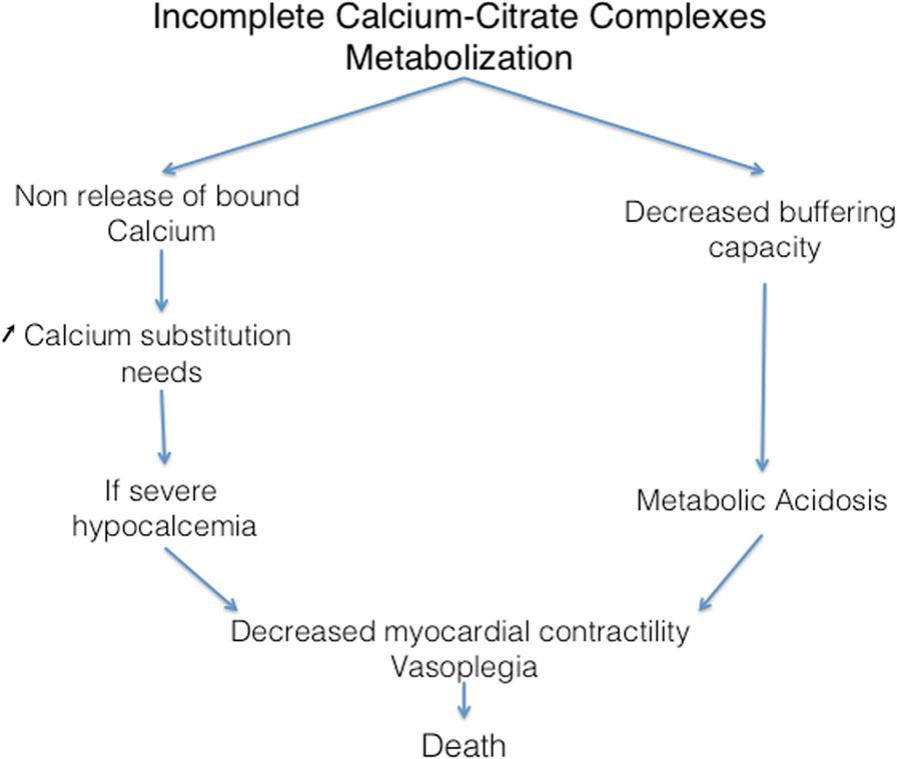
Consequences of citrate accumulation

Other signs are commonly observed during citrate accumulation. These signs should not be considered as diagnostic criteria but represent warning signs of potential citrate accumulation. Of those, an increase in calcium substitution needs might suggest the absence of CCC-bound calcium release and should prompt particular attention from clinicians. In overt citrate accumulation, hypocalcemia is usually observed, potentially leading to severe complications. Similarly, recurrence of high anion gap metabolic acidosis and increased serum lactate levels are also frequently observed concomitant to citrate accumulation. These anomalies are not thought to be secondary to citrate accumulation itself but rather to a common primary process impairing the tricarboxylic acid cycle, reducing citrate metabolism, and limiting pyruvate metabolism leading to lactate generation. Accumulated CCC participate in the elevated anion gap as well as to a strong ion gap. Pathophysiologic consequences of CCC accumulation are presented in Fig. 5.

#### Net citrate overload

Net citrate overload is a common, benign, and easy to manage complication of RCA. Citrate overload is a situation in which the organism’s capacity to metabolize citrate is *not* reached and all citrate–calcium complexes *are* metabolized (Fig. 4). The concomitant net load of sodium ions leads to plasma alkalinization through an increased SID. No increase in total/ionized calcium is observed and ionized calcium levels remain normal. Net citrate overload is a sign of excessive citrate administration or, more frequently, of low clearance in the hemofilter.

#### Insufficient trisodium-citrate delivery

Insufficient trisodium-citrate delivery is a situation where the alkalotic load administered to the patient is insufficient to adequately buffer acute kidney injury-associated acidosis, resulting in residual metabolic acidosis. This may happen if blood flow is set too low proportional to dialysate flow.

In this situation, the observed metabolic acidosis should not be interpreted as resulting from citrate accumulation. On the contrary, the adequate response should be to *increase* the blood flow or to *decrease* the dialysate flow. Key elements here are the normal total/ionized calcium ratio and calcium substitution rate.

### Situations at risk of citrate accumulation or net overload

Some situations lead to increased citrate delivery or decreased metabolizing capacity. According to the extent of this process, and the patient’s ability to metabolize CCC, it might lead to citrate accumulation or net overload (Fig. 4).

#### Excess citrate delivery to the patient

Accidental excess citrate infusion might occur in cases of incorrect circuit setup (e.g.,post-filter citrate administration) or administration of citrate when the blood pump is stopped. These issues are now unlikely with new generation CRRT devices with built-in citrate modules designed to prevent handling errors and increase safety. In particular, citrate administration is coupled with the blood pump. Such devices make use of specific tubes and connections as well as color codes, minimizing the risk of errors during circuit setup and use.

Citrate removal at the hemofilter level can be impaired, resulting in excess citrate delivery to the patient. Such an issue might occur in CVVH mode when the ultrafiltration rate is set too low or in CVVHD mode when an insufficient dialysate rate is set. These complications should be prevented by adherence to a strict protocol. Rapid loss of clearance at the filter level is occasionally observed in some patients with early clogging of the membranes. In such situations, the patient’s citrate delivery is higher than expected by the mathematical model driving the pumps and overload might occur. In this case, rapid replacement of the circuit is necessary. Of note, such a situation is unlikely to occur in CVVH mode, since early clogging would be identified by an increased transmembrane pressure.

Most of these situations may be prevented by medical and nursing education and their frequency should decrease with increasing experience.

#### Decreased citrate metabolization

In some situations citrate metabolization is decreased (Fig. 4b). A patient’s capacity to metabolize citrate is a dynamic process depending on baseline characteristics and hemodynamic status as well as mitochondrial function. Therefore, such situations are difficult to predict a priori, but some groups of patients should be considered at risk.

Patients with *acute liver failure* or acute-on-chronic liver failure have classically been described as having decreased citrate metabolizing capacity. However, recent literature has suggested that most patients in these situations could process citrate anyway and that classic markers of liver function were poor predictors of the risk of citrate accumulation [21–23]. As depicted in Fig. 4b, the ability of these patients to metabolize citrate is not *null* but simply decreased. Therefore, a protocol associated with low citrate delivery (normal or mildly reduced doses associated to increased clearance) to the patient is likely to be tolerated in most situations.

Patients with *circulatory shock* are likely to have a decreased oxygen delivery to the cells with decreased Krebs cycle activity due to reduced activity of the mitochondrial oxidation chain. Similarly, some commonly encountered intoxications (biguanides (e.g., metformin), cyclosporine, paracetamol, trichloroethylene, or propofol) can lead to mitochondrial blockage and decrease citrate metabolization capacity [24]. In these situations, a transient decrease in citrate metabolizing capacity is likely.

Since all these situations are typically associated with elevated serum lactate levels, such measurement is an important indicator of the body’s capacity to metabolize citrate. However, the lactate threshold above which RCA should not be used remains to be determined.

### Management

When either citrate accumulation or overload is suspected, the net citrate load finally administered to the patient must rapidly be decreased. According to the protocol used, this can be obtained either by 1) decreasing the blood flow rate (decreases intake through blood flow–citrate coupling) or 2) increasing the dialysate rate (CVVHD) or the filtration rate (CVVH) (increases removal), or 3) decreasing the targeted citrate concentration within the filter.

The two situations largely differ in potential severity and consequences. Citrate accumulation usually occurs in very severely ill patients. Unless rapid improvement after citrate delivery decrease is observed, RCA should be replaced by alternative circuit anticoagulation. Of note, in this situation, CRRT should be continued to enable CCC clearance. On the other hand, citrate overload is a benign process and should not prompt therapy discontinuation. It is usually fixed with citrate delivery reduction. Normalization of the pH, however, is a slow process and its correction requires time.

### Minimizing the risk of citrate accumulation

A strict protocol for RCA should be applied in all centers for all patients. Particular care should be taken in patients with suspected decreased citrate metabolization capacity (acute liver failure, circulatory shock, and intoxications). In centers with limited experience with the technique, RCA should probably be considered as contraindicated in such patients.

In addition to close monitoring of ionized calcium (post-filter for efficacy and systemic for safety), regular assessments of total/ionized Ca^2+^ and pH must be performed.

In general, well-designed protocols should aim to minimize citrate delivery to patients. This goal can be achieved by combining several measures:

1. Limited blood flow should be used. Indeed, since citrate administration is coupled to blood flow, lower blood flow means less need for citrate. This can easily be achieved in diffusion-based modes. Of note, in diffusive modes, low blood flows do not translate into low blood purification for two reasons: 1) dialysate rate remains the limiting factor and 2) high flux membranes are preferred for RCA, allowing important clearance even with reduced blood flows. Most protocols using diffusive modes would recommend blood flow between 80 and 150 ml/min. Purely convective techniques can be used but with a higher risk of metabolic complications. Indeed, the combination of low blood flows (to limit citrate administration) and high filtration rates (to optimize CCC clearance) would lead to high filtration fraction, increasing the risk of membrane clogging and decreased CCC clearance. This issue can be minimized if diluted citrate solutions are used as predilution.
2. High dialysate/filtration rates should be favored to increase citrate removal.

## Conclusions

Based on recent recommendations, the use of RCA is likely to increase dramatically throughout the world. RCA protocols should aim to minimize the amount of net citrate load delivered to the patient. In case of acid-base derangement during RCA, clinicians should be able to differentiate a benign citrate net overload (alkalosis) from a life-threatening accumulation (elevated Ca/Ca^++^, increase need for calcium substitution, and trend toward acidosis), which should prompt therapy tuning and early termination. The distinction between these two entities is crucial to enable adequate patient surveillance while receiving RCA.

## Abbreviations

ACD: Acid citrate dextrose
CCC: Citrate–calcium complexes
CRRT: Continuous renal replacement therapy
CVVH: Continuous veno-venous hemofiltration
CVVHD: Continuous veno-venous hemodialysis
CVVHDF: Continuous veno-venous hemodiafiltration
RCA: Regional citrate anticoagulation
SID: Strong ion difference
